# Differential relationships between autistic traits and anthropomorphic tendencies in adults and early adolescents

**DOI:** 10.3389/fpsyg.2024.1281207

**Published:** 2024-06-05

**Authors:** Rui-Rui Gao, Shang-Wen Si, Xiao-Xiao Lin, Yu-Zheng Wang, Ning Wang, Jin-Yan Wang, Fei Luo

**Affiliations:** ^1^Key Laboratory of Mental Health, Institute of Psychology, Chinese Academy of Sciences (CAS), Beijing, China; ^2^Department of Psychology, University of Chinese Academy of Sciences, Beijing, China; ^3^Beijing 101 Middle School, Beijing, China

**Keywords:** anthropomorphism, autistic traits, theory of mind, loneliness, sociality motivation

## Abstract

Anthropomorphism, the attribution of human-like qualities (e.g., mental states) to nonhuman entities, is a universal but variable psychological experience. Adults with professionally diagnosed autism or high levels of subclinical autistic traits consistently show greater tendencies to anthropomorphize, which has been hypothesized to reflect 1) a compensatory mechanism for lack of social connectedness and 2) a persistence of childhood anthropomorphism into adulthood. Here, we directly tested these hypotheses in a general population sample consisting of both adults (N=685, 17–58 years old) and early adolescents (N=145, 12–14 years old) using the refined 9-item Anthropomorphism Questionnaire (AnthQ9), which measures both present and childhood anthropomorphic tendencies. We found that adults with heightened autistic traits reported increased present anthropomorphism (e.g., tend more to perceive computers as having minds), which held even after controlling for social connectedness. In contrast, adolescents with heightened autistic traits did not show increased present anthropomorphism, but rather reported reduced childhood anthropomorphism (e.g., less likely to perceive toys as having feelings) after controlling for social connectedness. We also found evidence that the present and childhood subscales of the AnthQ9 may tap into fundamentally different aspects of anthropomorphism. The results suggest that increased anthropomorphic tendencies in adults with heightened autistic traits cannot be explained solely by increased sociality motivation, but may be due to delayed development of anthropomorphism, although alternative possibilities of measurement problems cannot be ruled out. Implications for the measurement of anthropomorphism and its relation with theory of mind were also discussed.

## Introduction

Anthropomorphism is the attribution of human-like qualities to non-human agents and objects (e.g., perceiving a doll locked in a box as feeling lonely). Anthropomorphism goes beyond animism (i.e., attributing life to the nonliving), as the perception of nonhuman entities as having minds or mental states is central to anthropomorphism (Epley et al., 2007, 2008). There is an ongoing debate about whether anthropomorphism involves mental processes and neural mechanisms similar to theory of mind (ToM), the attribution of mental states to other humans (Epley et al., 2007; Hortensius et al., 2021). The study of individual differences in anthropomorphism and its relation to ToM provides a unique opportunity to understand why we represent some entities but not others as mental agents, which is fundamental to social-cognitive processes (Frith and Frith, 2010; Waytz et al., 2010b).

Autism spectrum condition (ASC) is a neurodevelopmental condition characterized by differences in social interaction and communication and repetitive behaviors and/or restricted interests (American Psychiatric Association, 2022). According to the dimensional view of autism, ASC may represent the upper extreme of autistic traits that are continuously distributed in the population (Happé and Frith, 2020). ASC and subclinical autistic traits in the general population not only form a smooth continuum at the behavioral level (e.g., Baron-Cohen et al., 2001b), but also share common genetic etiologies (Lundström et al., 2012; Colvert et al., 2015). Many social-cognitive difficulties that characterize ASC, such as reduced ToM skills, are also present in typically developing individuals with high levels of autistic traits (Baron-Cohen et al., 2001a; Gökçen et al., 2016).

As both ToM and anthropomorphism involve the attribution of mental states to other entities, one might expect ASC (and autistic traits in general) to be associated with reduced anthropomorphic tendencies (Epley et al., 2007). In social cognition paradigms such as the Frith-Happé animation test, autistic individuals are less likely to describe animated geometric shapes that commonly elicit attributions of mental states in anthropomorphic terms and often describe them in purely mechanical terms (Abell et al., 2000; Klin, 2000; Castelli et al., 2002), which is often interpreted as supporting this view (e.g., Epley et al., 2007). Conversely, other researchers, informed by personal accounts of autistic authors such as Temple Grandin that they identify with nonhuman social agents such as animals, argue that anthropomorphic tendencies are intact or even enhanced in ASC (Atherton and Cross, 2018). Indeed, autistic individuals or individuals with subclinical autistic traits are equally or better able to reason the mental states of nonhuman targets, such as cartoon characters and animals, in anthropomorphic versions of ToM tasks (Atherton and Cross, 2019, 2022; Cross et al., 2022). However, the above studies have limited implications for the relationship between autistic traits and anthropomorphism, as the paradigms used are more concerned with whether typical social cues of mental states (e.g., animated movements and facial expressions) would elicit the perception of mind in autistic individuals, but do not speak to their spontaneous anthropomorphic tendencies in general.

More recent studies have directly measured anthropomorphism using self-report questionnaires, which consistently show increased anthropomorphic tendencies in adults with ASC (White and Remington, 2019; Atherton et al., 2023) or high levels of autistic traits (Tahiroglu and Taylor, 2019; Caruana et al., 2021; Clutterbuck et al., 2022). However, it is unclear what drives this association. A frequently cited hypothesis is that anthropomorphism may function as a compensatory mechanism to fulfill the unmet need for social connection with other humans in those with ASC or subclinical autistic traits, as they experience greater loneliness (Caruana et al., 2021). Sociality motivation has been argued to be one of the three major psychological determinants underlying individual differences in anthropomorphism (Epley et al., 2007), and an experimentally induced sense of social disconnection/connection can boost/attenuate people’s tendency to anthropomorphize (Epley et al., 2008; Bartz et al., 2016). Consistent with this hypothesis, Caruana et al. (2021) found that greater loneliness was associated with greater anthropomorphic tendencies in a sample of autistic adults, but they did not include non-autistic controls or assess autistic traits in this sample. Similarly, Atherton et al. (2023) found that autistic individuals endorsed a higher proportion of anthropomorphic versus physical descriptions of their pets than neurotypical individuals, but they did not examine this association after controlling for loneliness. Hence, it is unclear from these studies whether the association between autistic traits and anthropomorphism can solely be accounted for by loneliness, or conversely, whether autistic traits and loneliness may, at least in part, independently contribute to anthropomorphism.

Another possible reason, as implicitly suggested by White and Remington (2019), is that childhood anthropomorphism does not decline in autistic individuals as it does in typically developing individuals, but rather persists into adulthood. If this is the case, then the association between autism and anthropomorphism should emerge only in adulthood, not earlier in life. Indeed, using the Anthropomorphism Questionnaire (AnthQ; Neave et al., 2015), White and Remington (2019) found that autistic adults scored higher on the *Present Anthropomorphism subscale* but did not differ from non-autistic adults on the *Childhood Anthropomorphism subscale*, which has also been replicated in a general population sample (Clutterbuck et al., 2022; but see Caruana et al., 2021). However, given the retrospective nature of the Childhood Anthropomorphism subscale, this possibility requires further investigation in different age groups.

To address these issues, the present study replicated and extended previous studies to directly test the following two hypotheses in general population samples consisting of both adults and early adolescents. The first hypothesis (H1) was that the positive association between autistic traits and anthropomorphic tendencies in adults is driven by increased loneliness and social disconnection associated with high levels of autistic traits. The second hypothesis (H2) was that this association is driven by persistence of childhood anthropomorphism into adulthood in those with high levels of autistic traits. If H1 is correct, then the relationship between autistic traits and anthropomorphism should be largely accounted for by loneliness and other measures of social connectedness. If H2 is correct, then early adolescents with high and low levels of autistic traits should not differ (or at least differ less than adults) on anthropomorphism, as the difference should only emerge later in life.

## Method

### Participants

The adult sample consists of a college student sub-sample (S1, *N* = 254) and two online sub-samples (S2, *N* = 217; S3, *N* = 214) recruited separately for a larger research project on the relationship between social cognitions and autistic traits in the Chinese general population (total *N* = 685, 333 females, 17–58 years old). The adolescent sample consists of 167 early adolescents recruited from five classes in a middle school, with parent-report autistic traits provided by their parents. Twenty-two adolescents were removed due to missing values on the 9-item Anthropomorphism Questionnaire (AnthQ9) or the Autism Spectrum Quotient-10 (AQ-10), resulting in a final sample size of 145 (62 females, 12–14 years old). All adults provided online consent before their participation. For adolescents, their assent and parental consent were obtained prior to the study. All studies reported here were approved by the Research Ethics Committee of [Redacted] (H22017).

### Measures

#### Autistic traits

Autistic traits were measured in all participants using the Chinese version of the *Autism Spectrum Quotient-10 (AQ-10)* (Allison et al., 2012). Initially developed as a quick screener of potential autism, the AQ-10 has also been used as a measure of autistic traits in the general population (e.g., Greenberg et al., 2018). The possible range of the AQ-10 is 0–10, with higher scores indicating higher levels of autistic traits. In the present study, adults completed the self-report adult version of the AQ-10; for adolescents, their parents completed the parent-report adolescent version of the AQ-10. Both the adult and adolescent versions of the AQ-10 have shown good criterion validity but poor internal consistency in Chinese populations (Leung et al., 2023). We use McDonald’s omega instead of Cronbach’s alpha according to recent recommendations (Hayes and Coutts, 2020), as omega is considered a more general form of measure of reliability. An advantage of omega is that compared to alpha, it is less affected by the number of items. It is also noteworthy that in the present study, the questionnaires show similar levels of reliability regardless of whether omega or alpha coefficients were used. In the present study, the internal consistency was acceptable for the adolescent version (McDonald’s *ω* = 0.76) but poor for the adult version (McDonald’s *ω* = 0.32). Similarly poor internal consistency has also been found for the original adult version of the AQ-10, presumably because it is not essentially unidimensional (Taylor et al., 2020). Nevertheless, we chose to use the AQ-10 to allow comparison with previous studies of the relationship between autistic traits and anthropomorphism, most of which have used the AQ-10 (e.g., Clutterbuck et al., 2022).

To ensure the robustness of the results, we also administered the Chinese version of the *Broad Autism Phenotype Questionnaire (BAPQ)* (Hurley et al., 2007) in a sub-sample of adults (S1, *N* = 254), a measure of autistic traits with good internal consistency (overall McDonald’s *ω* = 0.90; subscale McDonald’s *ω*s = 0.88, 0.82, and 0.67 for social aloofness, pragmatic language, and behavioral rigidity, respectively) and acceptable convergent validity with the AQ-10 (Spearman’s *ρ* = 0.43, *p* < 0.001) in the present study.

#### Anthropomorphic tendencies

All participants completed a self-translated Chinese version of the 9-item refined version of the *Anthropomorphism Questionnaire (AnthQ9)* (Neave et al., 2015; Clutterbuck et al., 2022). Unlike another widely used anthropomorphism measurement, the Individual Differences in Anthropomorphism Questionnaire (IDAQ, Waytz et al., 2010a), which requires abstract thinking about whether common objects have minds, the AnthQ was designed to tap into the more intuitive aspects of anthropomorphism (i.e., people’s intuitive anthropomorphic thoughts and behaviors towards personal objects in daily lives) (Neave et al., 2015). The original AnthQ has 20 items, with 10 items designed to assess adult/present anthropomorphism (e.g., “On occasions I feel that my computer/printer/smartphone is being deliberately awkward”)^1^ and another 10 designed to assess childhood anthropomorphism (e.g., “As a child, when I put away my toys, I made sure that any odd ones lying around were placed with the others so that they would not feel lonely”). Clutterbuck et al. (2022) then refined the AnthQ into 9 items, with 4 items assessing adult/present anthropomorphism and 5 items assessing childhood anthropomorphism, which has a clearer two-factor structure. The AnthQ9 has shown improved psychometric properties and measurement invariance in people with different levels of autistic traits compared with the original version (Clutterbuck et al., 2022). ^2^

In the present study, our self-translated AnthQ9 had good internal consistency in both the adult (overall McDonald’s *ω* = 0.81; subscale McDonald’s *ω*s = 0.78 and 0.85 for *adult/present* and *childhood* anthropomorphism, respectively) and adolescent samples (overall McDonald’s *ω* = 0.85; subscale McDonald’s *ω*s = 0.71 and 0.88). In addition, our self-translated AnthQ9 has shown moderate to medium correlations with the adult and child versions of the IDAQ (*r*s = 0.32–0.51), demonstrating its convergence validity.

#### Social connectedness

Due to time constraints, a subset of the adult sample (S2, *N* = 217) completed measures of subjective loneliness and objective social connectedness. Loneliness was measured with the 8-item Chinese version of the UCLA Loneliness Scale (ULS-8), a measure widely used in Chinese-speaking adults (Wu and Yao, 2008) as well as adolescents (Xu et al., 2018). The ULS had good internal consistency in this adult sample (McDonald’s *ω* = 0.79). Objective social connectedness was measured by the “number of social ties” index of the Social Network Index (Cohen et al., 1997), which measures the total number of people with whom a respondent has regular contact (at least once every 2 weeks).

As for the adolescent sample, all of them reported subjective loneliness on the ULS-8 (McDonald’s *ω* = 0.76). We also assessed how frequently an adolescent was nominated as the best friend by their classmates using peer nomination procedure, which was used as a proxy of objective social connectedness.

#### Other measures of sociality motivations

It has been theorized that the need to long, the desire for acceptance and belongingness, is related to but distinct from the desire for mere social connectedness (Leary et al., 2013). Therefore, a subset of the adult sample (S3, *N* = 214) completed the 10-item Need to Belong Scale (NTBS; Leary et al., 2013; McDonald’s *ω* = 0.75). Because a strong need to belong can be associated with an increase in both preference for affiliation and fear of rejection, following Leary et al. (2013), we also administered in this sample the Preference for Affiliation (11 items; McDonald’s *ω* = 0.80) and the Fear of Criticism and Rejection (16 items; McDonald’s *ω* = 0.85) subscales from the Sociotropy dimension of the Sociotroypy-Autonomy Scale (Bieling et al., 2000).

### Statistical analysis

Datasets generated in the present study are openly available at https://osf.io/sdyba/files/osfstorage. All analyses except for mediation analyses were conducted with JASP 0.17 (JASP Core Team, 2023). Mediation analyses were performed by the Process (version 4.2) macro for SPSS (Hayes, 2012). Missing data were handled with listwise deletion.

Firstly, Spearman Rho (ρ) correlation coefficients were calculated between the AQ-10 and total anthropomorphism scores, present anthropomorphism, and childhood anthropomorphism, and then the same calculations as above were performed using the BAPQ (another questionnaire measuring autistic traits). We then tested the correlation of loneliness with autistic traits and anthropomorphism.

Secondly, our main hypotheses were examined using correlation analysis and multiple linear regressions. Age and sex were entered in all models following previous studies (Clutterbuck et al., 2022). To replicate previous findings on the association between autistic traits and anthropomorphism in adults, multiple linear regressions were conducted for the full adult sample with total scores of the AnthQ9, adult/present anthropomorphism, and childhood anthropomorphism as dependent variables, respectively, with age, sex, and autistic traits (AQ-10 scores) as predictors. We then tested whether this association was robust across different measures of autistic traits in adults (subsample S1) by substituting the AQ-10 with the BAPQ in the regression models, as the BAPQ demonstrated better reliability than the AQ-10.

In order to further elucidate whether social connectedness and other sociality motivations would account for the relationship between autistic traits and anthropomorphism (H1) in adults by entering variables on social connectedness (subsample S2) or other sociality motivations (subsample S3). As recommended by the reviewers, we performed supplemental mediation analyses with robust bootstrap test with 5,000 resamples using autistic traits as predictor, social connectedness variables as mediators, and childhood and present anthropomorphism as outcome variables. Finally, we tested whether higher levels of autistic traits would also be associated with increased anthropomorphism in adolescents (H2) by applying the same analyses procedures. Adjusted *R*^2^ is reported as indication of model fit. The alpha level was set to 0.05 for all analyses. In order to better clarify the issues examined by the different samples and the measurements used, we have created the following table of study summary.

Study summary: Why higher levels of autistic traits are associated with increased anthropomorphism?

**Table:** 

Sample		Measures	Research questions
Adult sample (*N*=)	S1 (*N* = 254)	Autistic traits: AQ10, BAPQ Anthropomorphism: AnthQ	Is this association robust across different assessments of autistic traits?
	S2 (*N* = 217)	Autistic traits: AQ10 Anthropomorphism: AnthQ Social connectedness: loneliness, social network size and diversity	Is this association driven by the lack of social connectedness?
	S3 (*N* = 214)	Autistic traits: AQ10 Anthropomorphism: AnthQ Sociality motivations: need to belong, preference for affiliation, fear of rejection	Is this association driven by other sociality motivations?
Adolescent sample (*N* = 145)		Autistic traits: AQ10 Anthropomorphism: AnthQ, IDAQ Social connectedness: loneliness, peer nominations	Is this association present in adolescence?

## Results

Sample demographics and descriptive statistics are presented in Tables 1–3.

**Table 1:** Sample demographics and descriptive statistics of Study 1.

	Adults								Adolescents	
	Overall (*N* = 685)		S1 (*N* = 254)		S2 (*N* = 217)		S3 (*N* = 214)		(*N* = 145)	
	Mean	SD	Mean	SD	Mean	SD	Mean	SD	Mean	SD
Age, year	27.48	6.87	21.28	1.93	30.59	5.76	31.68	6.33	12.87	0.69
Female, N	333	–	100	–	122	–	111	–	62	–
AQ-10	3.42	1.65	3.45	1.66	3.37	1.69	3.43	1.60	3.90	2.34
AnthQ	14.53	6.73	16.66	6.55	12.68	6.62	13.87	6.38	13.64	8.11
AnthQ_Present	3.87	3.41	4.70	3.69	2.99	2.91	3.79	3.32	3.87	3.46
AnthQ_Childhood	10.66	4.91	11.97	4.48	9.69	5.16	10.08	4.80	9.77	6.24
BAPQ			2.96	0.54						
BAPQ_aloof			2.67	0.77						
BAPQ_pragmatic			2.72	0.66						
BAPQ_rigid			3.50	0.52						
ULS-8					7.28	3.95			7.39	4.55
Objective SC *					20.12	9.25			2.50	1.66
NTBS							36.41	5.24		
Sociotropy_FCR							48.92	9.43		
Sociotropy_PA							38.16	7.00		

AQ-10, autism spectrum quotient-10; AnthQ, anthropomorphism questionnaire, adult/present subscale and childhood subscale; ULS-8, UCLA loneliness scale; Objective SC, objective social connectedness; NTBS, need to belong scale; FCR, fear of criticism and rejection; PA, preference for affiliation; BAPQ, broad autism phenotype questionnaire, social aloofness, pragmatic language, and behavioral rigidity subscales. *In adults, objective SC was proxied by the number of social ties as assessed by the Social Network Index. In adolescents, objective SC was proxied by the number of times one was nominated as a best friend by classmates.

**Table 2:** Correlations between autistic traits, anthropomorphism, and loneliness in a sample of adults.

	Mean (SD)	Correlation				
Variables		2	3	4	5	6
1. AQ-10	3.42(1.65)	0.119**	0.271***	−0.007	0.429***	0.307***
2. AnthQ	14.53(6.73)		0.663***	0.880***	0.163**	0.076
3. AnthQ_Present	3.87(3.41)			0.257***	0.463***	0.224**
4. AnthQ_Childhood	10.66(4.91)				−0.118	−0.012
5. BAPQ	2.96(0.54)					
6. ULS-8	7.28(3.95)					

**p < 0.01; ***p < 0.001.

**Table 3:** Correlations between autistic traits, anthropomorphism, and loneliness in a sample of adolescents.

	Mean (SD)	Correlation			
Variables		2	3	4	5
1. AQ-10	3.90(2.34)	−0.091	0.037	−0.129	0.409***
2. AnthQ	13.64(8.11)		0.678***	0.923***	0.240**
3. AnthQ_Present	3.87(3.46)			0.374***	0.209*
4. AnthQ_Childhood	9.77(6.24)				0.190*
5. ULS-8	7.39(4.55)				

*p < 0.05, **p < 0.01, ***p < 0.001.

In the adult sample, significant positive correlations were found between AQ-10 scores and total anthropomorphism scores [*r*(683) = 0.119, *p* = 0.002] and present anthropomorphism [*r*(683) = 0.271, *p* < 0.001], and there was no correlation with childhood anthropomorphism [*r*(683) = −0.007, *p* = 0.853]. And the relationship between BAPQ scores and anthropomorphism was similar to that between the AQ-10 scores and anthropomorphism. Positive correlations were significant between BAPQ with total anthropomorphism score [*r*(252) = 0.163, *p* = 0.009], with present anthropomorphism [*r*(252) = 0.463, *p* < 0.001], and no correlation was found with childhood anthropomorphism [*r*(252) = −0.118, *p* = 0.06].

Significant positive correlation between the scores of loneliness and the AQ-10 [*r*(215) = 0.307, *p* < 0.001], a significant positive correlation between loneliness scores and present anthropomorphism [*r*(215) = 0.224, *p* = 0.001], and no significant correlation with anthropomorphism total scores or childhood anthropomorphism [total scores: *r*(215) = 0.076, *p* = 0.262; childhood: *r*(215) = 0.012, *p* = 0.864]. Detailed information can be found in Table 2.

As for adolescent sample, no correlations were found between autistic trait scores and anthropomorphic total scores, present anthropomorphism, or childhood anthropomorphism [total score: *r*(143) = −0.091, *p* = 0.277; present: *r*(143) = 0.037, *p* = 0.658; childhood: *r*(143) = −0.129, *p* = 0.123]. Significant positive correlations were found between loneliness and autistic traits in the adolescent population [*r*(143) = 0.409, *p* < 0.001], and anthropomorphism total score, present anthropomorphism, and childhood anthropomorphism [total score: *r*(143) = 0.240, *p* = 0.004; present: *r*(143) = 0.209, *p* = 0.012; Childhood: *r*(143) = 0.190, *p* = 0.022]. Detailed information is available in Table 3.

### Anthropomorphism in adults

#### Association between anthropomorphism and autistic traits

We first attempted to replicate previous findings on the association between anthropomorphism and autistic traits in adults using the full adult sample. As shown in Table 4, after controlling for age and sex, AQ-10 scores had a weak but significant positive association with overall anthropomorphism (*β* = 0.10, *p* = 0.007). A closer inspection revealed that individuals with higher AQ-10 scores reported increased *present* (*β* = 0.10, *p* = 0.007) but not *childhood* anthropomorphism (*β* = −0.02, *p* = 0.538), suggesting that adults with heightened autistic traits had increased anthropomorphic tendencies at present but not in childhood.

**Table 4:** Association between autistic traits and anthropomorphism in adults.

Sample	DV	Adjusted *R*^2^	Predictor	*B*	SE	Beta	*t*	*p*
Adult all (*N* = 685)	AnthQ	0.047***	Age	−0.18	0.04	−0.19	−4.99	<0.001
			Sex	0.67	0.50	0.05	1.32	0.186
			**AQ-10**	**0.42**	**0.15**	**0.10**	**2.72**	**0.007**
	AnthQ Present	0.093***	Age	−0.08	0.02	−0.16	−4.35	<0.001
			Sex	0.69	0.25	0.10	2.77	0.006
			**AQ-10**	**0.49**	**0.08**	**0.24**	**6.43**	**<0.001**
	AnthQ Childhood	0.017**	Age	−0.10	0.03	−0.15	−0.39	<0.001
			Sex	−0.02	0.37	0.00	−0.07	0.948
			AQ-10	−0.07	0.11	−0.02	−0.62	0.538
Adult S1 (*N* = 254)	AnthQ	0.050***	Age	−0.29	0.21	−0.09	−1.38	0.170
			Sex	1.65	0.85	0.12	1.95	0.052
			**BAPQ_total**	**2.53**	**0.75**	**0.21**	**3.39**	**<0.001**
	AnthQ Present	0.252***	Age	−0.24	0.11	−0.13	−2.26	0.025
			Sex	1.48	0.42	0.20	3.49	<0.001
			**BAPQ_total**	**3.28**	**0.37**	**0.48**	**8.78**	**<0.001**
	AnthQ Childhood	−0.002, ns	Age	−0.05	0.15	−0.02	−0.35	0.725
			Sex	0.17	0.60	0.02	0.29	0.771
			BAPQ_total	−0.74	0.52	−0.09	−1.42	0.157

Significant non-demographic predictors were highlighted in bold font. **p < 0.01, ***p < 0.001, ns, not significant.

In addition to using AQ-10, we also used using the adult sub-sample S1, scores of the three subscales of the BAPQ to test the robustness of this pattern of associations, scores of the three subscales of the BAPQ were entered into the regression models in replacement of the AQ-10. As shown in Table 4, after controlling for age and sex, higher BAPQ total scores were significantly associated with greater overall (*β* = 0.21, *p* < 0.001) and *present* anthropomorphism (*β* = 0.48, *p* < 0.001) but not *childhood* anthropomorphism (*β* = −0.09, *p* = 0.157), suggesting that the association between autistic traits and present anthropomorphism in adults is robust regardless the measurement of autistic traits used. Unexpectedly, however, when the three subscales of the BAPQ were entered separately, only two subscales (*pragmatic deficits* and *rigid behaviors*) remained positively associated with overall and present anthropomorphism, whereas the *social aloofness* subscale was negatively associated with overall and childhood anthropomorphism (Table 5).

**Table 5:** Linear multiple regression models with BAPQ subscales.

Sample	DV	Adjusted *R*^2^	Predictor	*B*	SE	Beta	*t*	*p*
Adult S1 (*N* = 254)	AnthQ	0.124***	Age	0.03	0.22	0.01	0.14	0.890
			Sex	0.84	0.83	0.06	1.00	0.316
			**BAPQ_aloof**	**−2.34**	**0.70**	**−0.27**	**−3.32**	**0.001**
			**BAPQ_pragmatics**	**3.45**	**0.87**	**0.35**	**3.98**	**<0.001**
			**BAPQ_rigidity**	**2.02**	**0.93**	**0.16**	**2.16**	**0.032**
	AnthQ present	0.285***	Age	−0.12	0.11	−0.06	−1.06	0.290
			Sex	1.15	0.42	0.15	2.73	0.007
			BAPQ_aloof	−0.09	0.36	−0.02	−0.24	0.811
			**BAPQ_pragmatics**	**2.44**	**0.44**	**0.44**	**5.54**	**<0.001**
			**BAPQ_rigidity**	**0.97**	**0.48**	**0.14**	**2.03**	**0.043**
	AnthQ childhood	0.064***	Age	0.15	0.15	0.06	0.96	0.339
			Sex	−0.32	0.59	−0.04	−0.54	0.589
			**BAPQ_aloof**	**−2.25**	**0.50**	**−0.38**	**−4.52**	**<0.001**
			BAPQ_pragmatics	1.01	0.61	0.15	1.65	0.101
			BAPQ_rigidity	1.05	0.66	0.12	1.59	0.113

Significant non-demographic predictors were highlighted in bold font. **p<0.01, ***p<0.001.

#### Effects of social connectedness and other sociality motivations

To test H1, we controlled for effects of social connectedness by entering loneliness (ULS-8) and number of social ties (as assessed by the SNI) into the model using the adult sub-samples S2. As shown in Table 6, AQ-10 scores remained positively associated with overall (*β* = 0.15, *p* = 0.028) and *present* anthropomorphism (*β* = 0.24, *p* < 0.001) but not *childhood* anthropomorphism (*β* = 0.06, *p* = 0.378), suggesting that in adults, autistic traits were related to present anthropomorphic tendencies independent of social connectedness. Furthermore, as expected, greater loneliness was also associated with greater present anthropomorphism (*β* = 0.21, *p* = 0.003), suggesting that those who felt lonely would tend more to anthropomorphize. Unexpectedly, however, larger number of social ties was associated with greater rather than reduced overall (*β* = 0.29, *p* < 0.001) and childhood anthropomorphism (*β* = 0.31, *p* < 0.001), suggesting that those with more social connections would also tend more to anthropomorphize, at least in their childhood.

**Table 6:** Effects of social connectedness and other sociality motivations in adults.

Sample	DV	Adjusted *R*^2^	Predictor	*B*	SE	Beta	*t*	*p*
Adult S2 (*N* = 217)	AnthQ	0.091***	Age	−0.19	0.08	−0.16	−2.35	0.020
			Sex	0.28	0.88	0.02	0.32	0.748
			**AQ-10**	**0.60**	**0.27**	**0.15**	**2.22**	**0.028**
			ULS-8	0.22	0.12	0.13	1.90	0.059
			**Social ties**	**0.20**	**0.05**	**0.29**	**4.09**	**<0.001**
	AnthQ present	0.115***	Age	−0.05	0.04	−0.10	−1.39	0.165
			Sex	0.33	0.38	0.06	0.87	0.384
			**AQ-10**	**0.41**	**0.12**	**0.24**	**3.50**	**<0.001**
			**ULS-8**	**0.15**	**0.05**	**0.21**	**2.97**	**0.003**
			Social ties	0.03	0.02	0.10	1.41	0.160
	AnthQ childhood	0.070***	Age	−0.14	0.06	−0.16	−2.21	0.028
			Sex	−0.05	0.69	−0.01	−0.07	0.943
			AQ-10	0.19	0.21	0.06	0.88	0.378
			ULS-8	0.07	0.09	0.06	0.78	0.437
			**Social ties**	**0.17**	**0.04**	**0.31**	**4.41**	**<0.001**
Adult S3 (*N* = 214)	AnthQ	0.157***	Age	−0.01	0.07	−0.01	−0.13	0.895
			Sex	−1.10	0.81	−0.09	−1.36	0.176
			AQ-10	0.23	0.26	0.06	0.88	0.381
			NTBS	0.03	0.09	0.02	0.30	0.766
			Sociotropy FCR	0.07	0.05	0.10	1.29	0.198
			**Sociotropy PA**	**0.32**	**0.07**	**0.35**	**4.48**	**<0.001**
	AnthQ present	0.117***	Age	−0.02	0.04	−0.05	−0.70	0.485
			Sex	0.12	0.43	0.02	0.29	0.775
			**AQ-10**	**0.37**	**0.14**	**0.18**	**2.61**	**0.010**
			NTBS	−0.04	0.05	−0.07	−0.84	0.401
			**Sociotropy FCR**	**0.10**	**0.03**	**0.28**	**3.66**	**<0.001**
			Sociotropy PA	0.03	0.04	0.07	0.88	0.380
	AnthQ childhood	0.175***	Age	0.02	0.05	0.02	0.32	0.747
			Sex	−1.23	0.60	−0.13	−2.03	0.043
			AQ-10	−0.13	0.20	−0.05	−0.69	0.494
			NTBS	0.07	0.07	0.08	1.00	0.317
			Sociotropy FCR	−0.03	0.04	−0.07	−0.88	0.379
			**Sociotropy PA**	**0.29**	**0.05**	**0.42**	**5.40**	**<0.001**

Significant non-demographic predictors were highlighted in bold font. ***p < 0.001.

We then controlled for other sociality motivations using the adult sub-sample S3 by entering need to belong (NTBS), fear of criticism and rejection (Sociotropy FCR), and preference for affiliation (Sociotropy PA) into the model. As shown in Table 6, higher AQ-10 scores remained positively associated with *present* anthropomorphism (*β* = 0.18, *p* = 0.010) but not *childhood* anthropomorphism (*β* = −0.05, *p* = 0.494), again suggesting that in adults, autistic traits were related to present anthropomorphic tendencies independent of sociality motivations. In addition, fear of rejection was associated with reduced present anthropomorphism (*β* = 0.28, *p* < 0.001), whereas preference for affiliation was associated with increased overall (*β* = 0.35, *p* < 0.001) and childhood anthropomorphism (*β* = 0.42, *p* < 0.001).

Mediation analyses showed that in adults, autistic traits had significant total effects (c = 0.51, 95% CI [0.29, 0.73], *p* < 0.001) and indirect effects on present but not childhood anthropomorphism through loneliness (ab = 0.10, 95% CI [0.02, 0.21], *R^2^* = 0.12, *F*_2, 214_ = 14.78, *p* < 0.001), such that heightened level of autistic traits was associated with increased loneliness (*a* = 0.73, 95% CI [0.43, 1.03], *p* < 0.001), which in turn was associated with increased present anthropomorphism (*b* = 0.14, 95% CI [0.04, 0.24], *p* = 0.005). Autistic traits had significant direct effects on present anthropomorphism (*c*’ = 0.41, 95% CI [0.0005, 0.18]) even after indirect effects were accounted for (see Supplementary Figure S1). There was no significant indirect effect through the number of social ties (Supplementary Tables S1, S2).

### Anthropomorphism in early adolescents

#### Association between anthropomorphism and autistic traits

To test H2, we first looked at whether there would be an association between anthropomorphism and autistic traits in early adolescents as in adults. As shown in Table 7, after controlling for age and sex, AQ-10 scores had no association with overall, present, or childhood anthropomorphism in adolescents (|*β*|s < 0.11, *p*s > 0.153), which seemingly supports the notion that the difference on anthropomorphic tendencies between those with high and low levels autistic traits only emerges later in life.

**Table 7:** Associations between autistic traits and anthropomorphism in adolescents.

Sample	DV	Adjusted *R*^2^	Predictor	*B*	SE	Beta	*t*	*p*
Adolescents (*N* = 145)	AnthQ	0.092***	Age	−0.31	0.95	−0.03	−0.32	0.748
			Sex	−5.18	1.31	−0.32	−3.94	<0.001
			AQ-10	−0.26	0.28	−0.08	−0.94	0.347
	AnthQ present	−0.010, ns	Age	−0.25	0.43	−0.05	−0.58	0.562
			Sex	−0.59	0.59	−0.08	−0.99	0.323
			AQ-10	0.04	0.12	0.03	0.30	0.767
	AnthQ childhood	0.133***	Age	−0.06	0.71	−0.01	−0.08	0.936
			Sex	−4.59	0.99	−0.37	−4.65	<0.001
			AQ-10	−0.30	0.21	−0.11	−1.43	0.154

***p < 0.001; ns, not significant.

#### Effects of social connectedness

We further tested whether there would be an association between anthropomorphism and autistic traits in early adolescents after individual differences in social connectedness was accounted for. Unexpectedly, after controlling for loneliness (ULS-8) and a proxy of objective social connectedness (i.e., the number of times one was nominated as a best friend by classmates), the AQ-10 scores became significantly associated with reduced overall (*β* = −0.19, *p* = 0.027) and childhood anthropomorphism (*β* = −0.20., *p* = 0.022) (Table 8), suggesting that adolescents with heightened autistic traits tended to have reduced anthropomorphic tendencies in childhood. In addition, loneliness was associated with increased overall, present, and childhood anthropomorphism (|*β*|s > 0.26, *p*s < 0.005).

**Table 8:** Effects of social connectedness in adolescents.

Sample	DV	Adjusted *R*^2^	Predictor	*B*	SE	Beta	*t*	*p*
Adolescents (*N* = 145)	AnthQ	0.162***	Age	−0.51	0.91	−0.04	−0.56	0.580
			Sex	−4.03	1.30	−0.25	−3.10	0.002
			**AQ-10**	**−0.67**	**0.30**	**−0.19**	**−2.23**	**0.027**
			**ULS-8**	**0.58**	**0.16**	**0.33**	**3.70**	**<0.001**
			Nominations	0.26	0.41	0.05	0.62	0.535
	AnthQ present	0.049***	Age	−0.32	0.42	−0.06	−0.77	0.443
			Sex	−0.12	0.59	−0.02	−0.21	0.836
			AQ-10	−0.14	0.14	−0.09	−1.01	0.314
			**ULS-8**	**0.23**	**0.07**	**0.30**	**3.23**	**0.002**
			Nominations	0.05	0.19	0.03	0.28	0.781
	AnthQ childhood	0.171***	Age	−0.19	0.70	−0.02	−0.27	0.789
			Sex	−3.90	0.99	−0.31	−3.93	<0.001
			**AQ-10**	**−0.53**	**0.23**	**−0.20**	**−2.31**	**0.022**
			**ULS-8**	**0.35**	**0.12**	**0.26**	**2.92**	**0.004**
			Nominations	0.20	0.32	0.05	0.65	0.518

Significant non-demographic predictors were highlighted in bold font. ***p < 0.001.

Mediation analyses showed that in adolescents, autistic traits had no significant indirect effects through loneliness on present anthropomorphism. In addition, neither total effects nor direct effects were significant. Autistic traits had significant indirect effects (ab = 0.35, 95% CI [0.14, 0.58], *R*^2^ = 0.10, *F*_2, 142_ = 8.32, *p* < 0.001) on childhood anthropomorphism through loneliness, such that heightened levels of autistic traits were associated with increased loneliness (*a* = 0.78, 95% CI [0.49, 1.08], *p* < 0.001), which in turn was associated with increased childhood anthropomorphism (*b* = 0.44, 95% CI [0.21, 0.68], *p* < 0.001). However, autistic traits also had significant direct effects on childhood anthropomorphism in the opposite direction (*c*’ = −0.70, 95% CI [−0.16, −0.24], *p* = 0.003), resulting in non-significant total effects (see Supplementary Figure S2). There was no significant indirect effect through the number of peer nominations (Supplementary Table S3).

## Discussion

Autism is characterized by atypicality in ToM abilities, which involves difficulty ascribing mental states to human agents (Happé and Frith, 2020; Lord et al., 2020). Intriguingly, recent studies have consistently shown that autistic adults or typically developing adults with high levels of autistic traits consistently report increased anthropomorphic tendencies, suggesting that they also tend to “overattribute” mental states to non-human agents or objects (Tahiroglu and Taylor, 2019; White and Remington, 2019; Caruana et al., 2021; Clutterbuck et al., 2022). Consistent with these studies, in a large adult sample, we found small to moderate positive associations between anthropomorphic tendencies and self-report autistic traits, which are robust regardless the measure of autistic traits used, as using both the AQ-10 and the BAPQ yielded a similar pattern of results. More importantly, the present study extended these findings by examining two possible reasons that may underlie this association.

The first hypothesis (H1) we examined is that adults with heightened autistic traits have increased anthropomorphic tendencies as a compensation for the lack of social connection with humans. Following the three-factor model of anthropomorphism (Epley et al., 2007) and the fact that autistic individuals tend to feel increasingly lonely, Caruana et al. (2021) argued that the association between autistic traits and anthropomorphism may be driven by an increase in sociality motivation due to loneliness and social isolation. In line with this notion, they found a positive association between loneliness and anthropomorphism in a sample of autistic adults (Caruana et al., 2021). In addition, in a qualitative study on the first-hand experience of anthropomorphism, autistic individuals expressed the importance of using anthropomorphism as a way to ease loneliness (Negri et al., 2019). A potential limitation of this line of work is that loneliness and autistic traits are not measured simultaneously: it is still possible that sociality motivation and autistic traits are independently related to anthropomorphism. In the present study, we found that the positive association between autistic traits and anthropomorphic tendencies in adults held even after social connectedness (i.e., subjective loneliness and number of social ties) and other measures of sociality motivation (need to belong, fear of rejection, and preference for affiliation) were controlled for. In other words, adults with higher levels of autistic traits would still tend more to anthropomorphize compared to those with fewer autistic traits even if they have same levels of loneliness. On the other hand, consistent with H1, the results from the mediation analyses indicated that autistic traits did have indirect effects on anthropomorphism through loneliness. However, in adults, even after controlling for these indirect effects, autistic traits still had significant direct effects on anthropomorphism, which is inconsistent with H1. Overall, these findings partially support H1, but also highlighted the fact that the association between autistic traits and anthropomorphism was not driven by loneliness or other sociality motivations alone.

The second hypothesis (H2) we examined is whether increased anthropomorphic tendencies in adults with heightened autistic traits reflect a persistence of their childhood anthropomorphism. Children generally have more anthropomorphic thoughts and behaviors than adults, and this childhood anthropomorphism typically declines during development (Epley et al., 2007). However, autistic adults do not differ from non-autistics on the *Childhood Anthropomorphism subscale* but have higher scores on the *Present Anthropomorphism subscale* of the AnthQ (White and Remington, 2019), suggesting that anthropomorphism may not decline during development in those with autism or heightened autistic traits. If this is the case, then the positive association between autistic traits and anthropomorphism should not emerge in early stage of life. Partially consistent with this hypothesis, in the present study, early adolescents with high and low levels of autistic traits did not differ in their *present* anthropomorphic tendencies. Unexpectedly, however, early adolescents with high levels of autistic traits showed reduced overall and childhood anthropomorphic tendencies after controlling for loneliness and objective social connectedness. This may suggest that the development of anthropomorphism is delayed in individuals with heightened subclinical autistic traits: they may begin to develop anthropomorphic representations later in childhood (as suggested by Epley et al., 2007), then keep up with individuals with fewer autistic traits in early adolescence, and eventually surpass them in adulthood when anthropomorphism has declined in those with fewer autistic traits. However, given the retrospective nature of the Childhood Anthropomorphism subscale and the fact that high-autistic-traits adults did not report reduced childhood anthropomorphism as adolescents did, further research in younger children would be needed to depict actual development trajectories of anthropomorphism in individuals with high and low levels of autistic traits.

Another important finding from the present study is that the two subscales of the AnthQ9 seem to tap into not just anthropomorphism in different time frames, but essentially different aspects of anthropomorphism. In the present study, the Present Anthropomorphism subscale is more consistently associated with more autistic traits, poorer social outcomes (e.g., increased loneliness), and avoidance social motivations (i.e., fear of rejection). On the contrary, across the adult and adolescent samples, the Childhood Anthropomorphism subscale is more consistently associated with fewer autistic traits or lower levels of social aloofness, better social outcomes (e.g., more social ties in adulthood), and approach social motivations (i.e., preference for affiliation). We suggest that the *present* subscale may tap into more egocentric and distressful anthropomorphic experiences, as its all four items involve perceiving digital devices/weather as being deliberately against oneself, and this kind of anthropomorphic experiences has been described as distressing by some autistic individuals (Negri et al., 2019). On the contrary, the *childhood* subscale may tap into more allocentric anthropomorphic experiences, as its all five items involve perceiving toys as having feelings and taking care of them. This kind of anthropomorphic experiences are often observed in pretend play behaviors in early childhood, which is viewed as a precursor of ToM development (Lillard, 1993; Weisberg, 2015) and is often absent in autistic children (Jarrold, 2003). Given that anthropomorphic experiences can be egocentrically biased (Epley et al., 2007) and the equivocal evidence regarding the relationship between anthropomorphism and ToM (Tahiroglu and Taylor, 2019; Hortensius et al., 2021), we suggest that future research should try to distinguish between the different aspects (i.e., egocentric vs. allocentric) of anthropomorphism and include measures of ToM to clarify the relationships among anthropomorphism, ToM, and autistic traits.

Several limitations of the present study should be noted. First, the adult version of the AQ-10 has poor internal consistency, and future research should seek to replicate the present findings using other measurements of autistic traits, such as the full AQ, the BAPQ, and the Subthreshold Autism Trait Questionnaire (SATQ) (Kanne et al., 2012). Second, the present study was based on participants recruited from general populations, which should not be generalized to clinically diagnosed autism. Third, it should be noted that not all adult participants completed the same measures except for autistic traits and anthropomorphism, as the three subsamples were recruited for separate purposes. In addition, our adolescent sample was smaller than the adult sample. We initially determined the target sample size for adolescents according to an *a priori* power analysis, which showed that a sample size of 133 would have sufficient power (*β* > 0.80) at the 0.05 alpha level to detect a medium effect (*ρ* = 0.24), an effect size observed in our adult sample (*ρ* > 0.24) and in some previous studies (e.g., Caruana et al., 2021; *ρ* > 0.25). However, we noted that this effect size could sometimes be quite small (e.g., Clutterbuck et al., 2022; *ρ* ≈ 0.10), which could mean that the adolescent sample might be underpowered. However, even if this was the case, it does not affect the conclusion that the association between autistic traits and anthropomorphism was much weaker in early adolescents than in adults, as this association was robustly detected in all three adult subsamples (*ρ*s > 0.24, *N*s = 254, 217, 214, respectively). Finally, we were unable to test H2 in younger children due to their difficulty in understanding some of the AnthQ9 items as they do not have regular access to personal computers/smartphones. Future studies should seek to modify these items for younger children.

## Conclusion

In summary, the present study shows that increased anthropomorphic tendencies in adults with heightened subclinical autistic traits cannot be viewed merely as a compensation for a lack of social connections, but may be due to delayed development of anthropomorphism, although alternative explanations should also be considered. In addition, the two subscales of the AnthQ9 seem to tap into essentially different aspects of anthropomorphism, which may have completely different relationships with ToM and should be taken into considerations in future research.

## Data availability statement

The datasets presented in this study can be found in online repositories. The names of the repository/repositories and accession number(s) can be found below: datasets generated in the present study are openly available at the project’s Open Science Framework page (https://osf.io/sdyba/files/osfstorage).

## Ethics statement

The studies involving humans were approved by Research Ethics Committee of Institute of Psychology, Chinese Academy of Sciences (H22017). The studies were conducted in accordance with the local legislation and institutional requirements. Written informed consent for participation in this study was provided by the participants’ legal guardians/next of kin.

## Author contributions

R-RG: Data curation, Investigation, Methodology, Project administration, Writing – original draft, Writing – review & editing. S-WS: Investigation, Project administration, Resources, Writing – review & editing. X-XL: Conceptualization, Funding acquisition, Project administration, Resources, Writing – review & editing. Y-ZW: Funding acquisition, Writing – review & editing. NW: Funding acquisition, Writing – review & editing. J-YW: Funding acquisition, Writing – review & editing. FL: Funding acquisition, Writing – review & editing.

## Footnotes

^1^The original item only asks about computers and printers. We added “smartphone” when translating the AnthQ9 because not all Chinese participants have regular access to personal computers and printers, but most of them (including adolescents) possess a smartphone.

^2^We further changed the original 7-point Likert scale to a 5-point scale from 0 (Not at all) to 4 (Very much so), as our pilot studies suggest that 7 grades are redundant.
