# Supplementary material for: Differential relationships between autistic traits and anthropomorphic tendencies in adults and early adolescents
Source: Front Psychol. 2024 Jun 5;15:1281207. doi: 10.3389/fpsyg.2024.1281207 (PMC11186442; doi:10.3389/fpsyg.2024.1281207)
Supplement: Supplementary file 1 [file Data_Sheet_1.docx]

Supplementary Material

# Supplementary Data

| TableS1 Mediating effects of social connectedness between autistic traits and anthropomorphism(Sample 2) | | | | | | | |
| --- | --- | --- | --- | --- | --- | --- | --- |
| Independent variable | Mediator variable | Outcome variable | Type of effect | effect value | Boot SE | 95% Confidence Interval | Percentage of effect |
| AQ-10 | ULS-8 | AnthQ_Present | Total effect | 0.51 | 0.11 | [0.29, 0.73] | 100% |
|  |  |  | Direct effect | 0.41 | 0.12 | [0.18, 0.64] | 80.39% |
|  |  |  | Indirect effect | 0.10 | 0.05 | [0.02, 0.20] | 19.61% |
|  |  | AnthQ_Childhood | Total effect | 0.16 | 0.21 | [-0.25, 0.57] | 100% |
|  |  |  | Direct effect | 0.163 | 0.22 | [-0.27, 0.60] | 101.88% |
|  |  |  | Indirect effect | -0.003 | 0.07 | [-0.14, 0.14] | -1.88% |
|  | Objective SC | AnthQ_Present | Total effect | 0.511 | 0.11 | [0.29, 0.73] | 100% |
|  |  |  | Direct effect | 0.518 | 0.11 | [0.29, 0.74] | 101.37% |
|  |  |  | Indirect effect | -0.007 | 0.02 | [-0.06, 0.03] | 1.37% |
|  |  | AnthQ_Childhood | Total effect | 0.16 | 0.21 | [-0.25, 0.57] | 100% |
|  |  |  | Direct effect | 0.27 | 0.20 | [-0.13, 0.67] | 168.75% |
|  |  |  | Indirect effect | -0.11 | 0.06 | [-0.24, -0.005] | -68.75% |

*Note.* AQ-10: Autism Spectrum Quotient-10; AnthQ: Anthropomorphism Questionnaire, adult/present subscale and childhood subscale; ULS-8: UCLA Loneliness Scale; Objective SC:the number of social ties as assessed by the Social Network Index

| TableS2 Mediating effects of social connectedness between autistic traits and anthropomorphic tendencies (Sample 3) | | | | | | | |
| --- | --- | --- | --- | --- | --- | --- | --- |
| Independent variable | Mediator variable | Outcome variable | Type of effect | Effect value | Boot SE | 95% Confidence Interval | Percentage of effect |
| AQ-10 | NTBS | AnthQ_Present | Total effect | 0.503 | 0.14 | [0.23,0.78] | 100% |
|  |  |  | Direct effect | 0.511 | 0.14 | [0.24, 0.78] | 101.59% |
|  |  |  | Indirect effect | -0.008 | 0.007 | [-0.02, 0.007] | -1.59% |
|  |  | AnthQ_Childhood | Total effect | -0.11 | 0.21 | [-0.52, 0.29] | -100% |
|  |  |  | Direct effect | -0.07 | 0.20 | [-0.47, 0.32] | -63.64% |
|  |  |  | Indirect effect | -0.04 | 0.05 | [-0.15, 0.06] | -36.36% |
|  | Sociotropy FCR | AnthQ_Present | Total effect | 0.5031 | 0.14 | [0.23, 0.78] | 100% |
|  |  |  | Direct effect | 0.3863 | 0.14 | [0.12, 0.65] | 76% |
|  |  |  | Indirect effect | 0.1168 | 0.05 | [0.04, 0.22] | 24% |
|  |  | AnthQ_Childhood | Total effect | -0.11 | 0.21 | [-0.52, 0.29] | -100% |
|  |  |  | Direct effect | -0.20 | 0.21 | [-0.61, 0.21] | -181.81% |
|  |  |  | Indirect effect | 0.09 | 0.06 | [-0.003, 0.24] | 81.81% |
|  | Sociotropy_PA | AnthQ_Present | Total effect | 0.50 | 0.14 | [0.23, 0.78] | 100% |
|  |  |  | Direct effect | 0.49 | 0.14 | [0.22, 0.76] | 98% |
|  |  |  | Indirect effect | 0.01 | 0.02 | [-0.04, 0.06] | 2% |
|  |  | AnthQ_Childhood | Total effect | -0.11 | 0.21 | [-0.52, 0.29] | -100% |
|  |  |  | Direct effect | -0.15 | 0.19 | [-0.52, 0.21] | -136.36% |
|  |  |  | Indirect effect | 0.04 | 0.08 | [-0.12, 0.21] | 36.36% |

*Note.* AQ-10: Autism Spectrum Quotient-10; AnthQ: Anthropomorphism Questionnaire, adult/present subscale and childhood subscale; ULS-8: UCLA Loneliness Scale; NTBS: Need To Belong Scale; FCR: Fear of Criticism and Rejection; PA: Preference for Affiliation

| TableS3 Mediating effects of social connectedness between autistic traits and anthropomorphism | | | | | | | |
| --- | --- | --- | --- | --- | --- | --- | --- |
| Independent variable | Mediator variable | Outcome variable | Type of effect | Effect value | Boot SE | 95% Confidence Interval | Percentage of effect |
| AQ-10 | ULS-8 | AnthQ_Present | Total effect | 0.03 | 0.12 | [-0.21, 0.28] | 100% |
|  |  |  | Direct effect | -0.15 | 0.13 | [-0.40, 0.11] | -500% |
|  |  |  | Indirect effect | 0.18 | 0.07 | [0.06, 0.34] | 600% |
|  |  | AnthQ_Childhood | Total effect | -0.35 | 0.22 | [-0.79, 0.08] | 100% |
|  |  |  | Direct effect | -0.70 | 0.23 | [-1.16, 0.24] | -200% |
|  |  |  | Indirect effect | 0.35 | 0.11 | [0.13, 0.58] | 100% |
|  | Peer Nominations | AnthQ_Present | Total effect | 0.0342 | 0.12 | [-0.21, 0.28] | 100% |
|  |  |  | Direct effect | 0.0082 | 0.13 | [-0.25, 0.27] | 23.98% |
|  |  |  | Indirect effect | 0.0259 | 0.04 | [-0.06, 0.13] | 76.02% |
|  |  | AnthQ_Childhood | Total effect | -0.3518 | 0.22 | [-0.79, 0.08] | 100% |
|  |  |  | Direct effect | -0.3753 | 0.24 | [-0.84, 0.09] | -106.68% |
|  |  |  | Indirect effect | 0.0236 | 0.08 | [-0.13, 0.21] | 6.68% |

*Note.* AQ-10: Autism Spectrum Quotient-10; AnthQ: Anthropomorphism Questionnaire, adult/present subscale and childhood subscale; Peer Nominations: the number of times one was nominated as a best friend by classmates


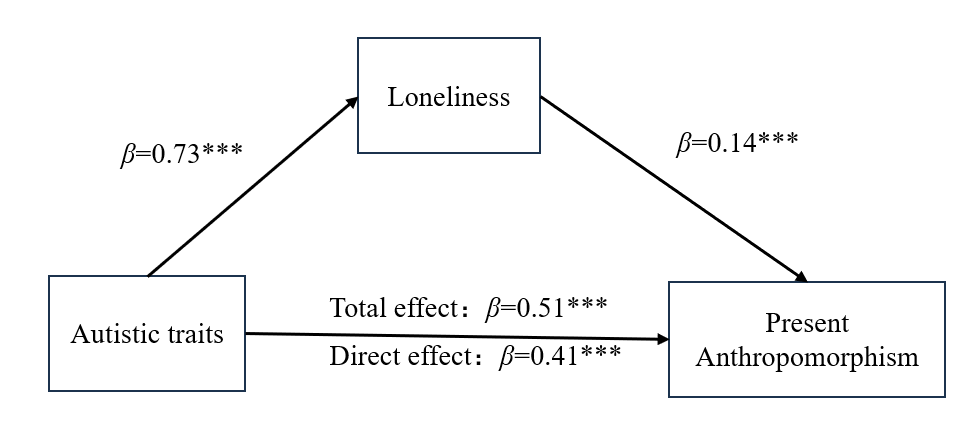


**Figure S1** Loneliness mediates autistic traits and present anthropomorphism pathways in adult Sample 2. The prediction coefficient (*β*) and significance are shown in the figure. ^***^ *p* < 0.001.


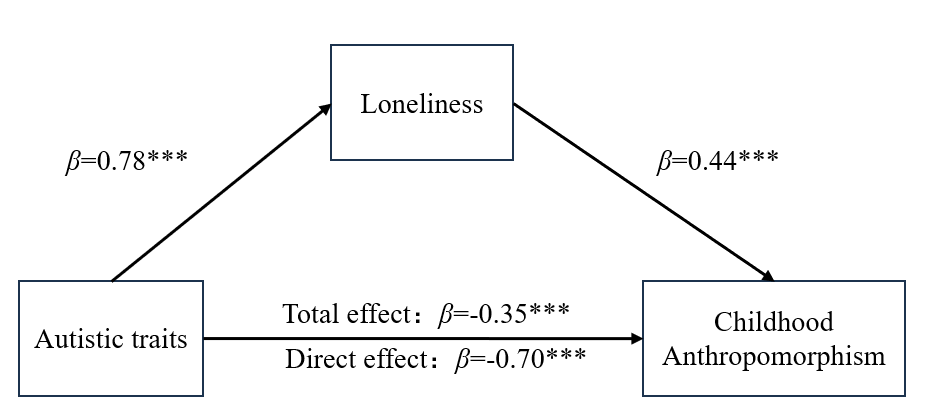


**Figure S2** Loneliness mediates autistic traits and childhood anthropomorphism pathways in adolescent sample. The prediction coefficient (*β*) and significance are shown in the figure. ^***^ *p* < 0.001.
